# Coagulation Parameters: An Efficient Measure for Predicting the Prognosis and Clinical Management of Patients with COVID-19

**DOI:** 10.3390/jcm9113482

**Published:** 2020-10-28

**Authors:** Manuel Quintana-Díaz, Eva María Andrés-Esteban, Karen Lizzette Ramírez-Cervantes, Bárbara Olivan-Blázquez, Raúl Juárez-Vela, Vicente Gea-Caballero

**Affiliations:** 1 Intensive Care Unit, La Paz University Hospital, Research Group BPM, Idi-Paz Research Institute, Paseo de la Castellana, 261, 28046 Madrid, Spain; mquintanadiaz@gmail.com; 2 Research Group BPM, Idi-Paz Research Institute, University Rey Juan Carlos, Calle de Pedro Rico, 6, 28029 Madrid, Spain; karenlizzetteramirez@gmail.com; 3 Research Unit in Primary Care, IIS Aragón, Avda, University of Zaragoza, San Juan Bosco, 13, 50009 Zaragoza, Spain; barbaraolivan@gmail.com; 4 Faculty of Health Sciences, La Rioja University, Edificio de Rectorado, Av. de la Paz, 93, 26006 Logroño, Spain; 5 Nursing School La Fe, Adscript Center of University of Valencia, Research Group GREIACC, Health Research Institute La Fe, Avda, Fernando Abril Martorell 106, Pabellón Docente Torre H, 46006 Valencia, Spain; gea_vic@gva.es

**Keywords:** coronavirus, COVID-19, SARS-CoV-2, coagulation

## Abstract

Background. COVID-19 is an ongoing global pandemic. Since the detection of the first cases of coronavirus disease 2019 (COVID-19) in Wuhan, China, the current pandemic has affected more than 25.3 million people worldwide. The aim of this study was to evaluate the relationship between coagulation abnormalities and prognosis in a cohort of patients with COVID-19. Methods. We performed a retrospective cohort study of 3581 patients admitted to Hospital La Paz (Madrid, Spain) due to respiratory infection by severe acute respiratory syndrome coronavirus from the beginning of the current pandemic to 15 July 2020. Results. Of the 3581 study patients, 48.94% were men, and 19.80% were healthcare workers. The median age was 62 years. Compared with the survivors, the non-survivors had lower prothrombin activity (82.5 (Interquartile range—IQR, 67–95) vs. 95.25 (IQR, 87–104) for non-survivors and survivors, respectively; *p* < 0.001), higher fibrinogen levels (748.5—IQR, 557–960) vs. 572.75 (IQR, 417–758; *p* < 0.001), and notably higher D-dimer levels (2329—IQR, 1086.12–5670.40) vs. 635.5 (IQR, 325.5–1194.8); *p* < 0.001). Conclusions. The evaluation of coagulation parameters could be an efficient measure for predicting the prognosis and improving the clinical management of patients with COVID-19.

## 1. Introduction

Since the detection of the first cases of coronavirus disease 2019 (COVID-19) in Wuhan, China, the current pandemic has affected more than 25.3 million people worldwide, reaching high rates of mortality in high-risk individuals and presenting multiple manifestations in addition to pulmonary complications [1,2,3]. Coagulation abnormalities are typical findings in patients with COVID-19 and are associated with poorer prognoses and survival. High D-dimer levels, for example, are consistently associated with poor outcomes and death [4]. Similarly, a significant prothrombin time (PT) prolongation has been observed in severe cases and is more evident among non-survivors [5]. During the acute response of the infection, fibrinogen levels can reach upper limits, which can persist as the disease progresses; however, a sudden decrease in these levels has been observed shortly before death in a patient cohort with novel coronavirus pneumonia [5,6]. The hematological abnormalities observed in COVID-19 patients suggest a procoagulant state that has been linked to both arterial and venous thrombosis, which is more frequently reported in severe cases [7,8,9]. A study conducted in China found that among 138 cases of COVID-19, venous thromboembolic events (VTE) occurred in 2.9%; however, in a smaller sample of critically ill patients admitted to intensive care units (ICUs), the VTE rate was 20% [10]. In France, pulmonary embolism (PE) rates in a single-center have been compared between ICU admissions for COVID-19 in 2020 and the general ICU population of the previous year, finding rates of 20.6% and 6.1%, respectively [11]. Coagulopathy has been reported in up to 50% of patients with severe COVID-19 manifestations, in whom disseminated intravascular coagulopathy (DIC) has been found in more than 70% of the cases [8]. However, the coagulation profile usually observed in DIC might not be consistent with the findings in COVID-19 patients, and the development mechanisms might differ [9]. In addition, a number of abnormal coagulation parameters, such as elevated D-dimer levels, have been found to be an independent risk factor for mortality for these patients [4].

Despite the numerous ongoing studies evaluating the underlying physiopathology of coagulopathy in COVID-19, it remains poorly understood [12,13]. Prophylactic therapy has been recommended for hospitalized patients because it appears to reduce mortality due to coagulation disorders [14]. In some cases, however, PE and venous VTE have been diagnosed in COVID-19 patients regardless of the administration of standard pharmacological thromboprophylaxis [11,15,16]. Anticoagulation might, therefore, be insufficient for certain circumstances, and alternative or additional therapies might be required.

Given that abnormal coagulation parameters might be associated with poor prognoses, monitoring hemostatic markers in all patients with COVID-19 might be advisable. The aim of this study was, therefore, to evaluate the relationship between coagulation abnormalities and prognosis in a cohort of hospitalized COVID-19 patients in a high-level hospital in Madrid, Spain.

We presented a retrospective analysis of 3581 patients to establish the relationship between coagulation parameters and poor outcomes in COVID-19 patients.

## 2. Methods

### 2.1. Study Design and Participants

We conducted a retrospective cohort study and analyzed the sociodemographic data, clinical status, laboratory test results, and medical management information during the hospitalization of 3581 patients admitted to La Paz University Hospital (Madrid, Spain) due to respiratory infection by severe acute respiratory syndrome coronavirus from the start of the current pandemic to the 15th of July 2020. The laboratory test results were obtained during the patients’ hospitalization and are presented as medians for all data collected during all processes and in all units. For the inclusion criteria, we analyzed all patients with COVID-19 based on the detection of SARS-CoV-2 RNA in throat swab specimens. The study was conducted in accordance with the principles of the Declaration of Helsinki, the Harmonized Tripartite Guidelines for Good Clinical Practice of the International Conference on Harmonization, the guidelines for Good Epidemiological Practice, and the European and Spanish regulations for the protection of personal data. The study was approved by the Clinical Research Ethics Committee of the La Paz University Hospital (HULP code: PI-4155).

#### 2.1.1. Main Variables

The main study variables were death, survival, and the need for ICU admission.

#### 2.1.2. Secondary Variables

The secondary variables included the patients’ sociodemographic data, previous medical history, clinical outcomes during hospitalization, and the following coagulation parameters recorded at admission and during hospitalization: PT in seconds and % of plasma dilution (prothrombin activity), international normalized ratio (INR), activated partial thromboplastin time (aPTT) with kaolin, aPTT with kaolin ratio, D-dimer, and fibrinogen.

### 2.2. Procedures and Statistical Analysis

All demographic, clinical, laboratory, treatment, and outcome data were extracted from the electronic records of La Paz University Hospital. Quantitative variables were presented using robust statistics, such as mean and interquartile ranges (IQRs), and qualitative data were presented using their frequency distribution. For the comparison of quantitative data between groups, we employed the Kruskal–Wallis non-parametric H test and the Shapiro–Wilk test for non-normally distributed data. For the comparison of qualitative variables, we employed the chi-squared test. We performed the survival estimates using the Kaplan–Meier method, comparing the survival curves according to the coagulation parameters between the groups using the Wilcoxon test, given that the survival curves did not reach the median survival. We constructed these curves according to the parameters considered in the range of normality by the hospital’s laboratory. We performed the multivariate analysis using a Cox regression with the forward conditional method, introducing the coagulation factors as the independent variables. The results of the multivariate model were presented as hazard ratios (95% confidence interval [CI]) and were graphically represented by a nomogram. 

## 3. Results

Of the 3581 study participants, 48.94% were men, and 19.80% were healthcare workers. The median age was 62 years (IQR, 47–78). Table 1 lists the patients’ demographic and clinical characteristics. In terms of the possible causes of transmission, 17.09% of the patients reported direct contact with an infected person, while 30.56% had suspected nosocomial COVID-19 infection.

Upon arrival at the emergency room, 63.22% of the patients required oxygen therapy, with nasal cannulas the most widely employed oxygen delivery device. Only 5.08% of the participants required ICU admission at arrival.

Table 2 shows the differences in blood coagulation parameters between the survivors and non-survivors. Despite the normal ranges, the non-survivors had a lower prothrombin activity (82.5—IQR, 67–95) vs. 95.25 (IQR, 87–104); *p* < 0.001), higher fibrinogen levels (748.5—IQR, 557–960) vs. 572.75 (IQR, 417–758); *p* < 0.001), and notably higher D-dimer levels (2329 (IQR, 1086.12–5670.40) vs. 635.5 (IQR, 325.5–1194.8); *p* < 0.001) than the survivors.

In terms of ICU admission as a poor prognosis factor, we observed that not all coagulation parameters were statistically different between the patients who were admitted to ICU and those not admitted (Table 3). Only prothrombin activity, PT, and D-dimer levels were associated with ICU admission. Prothrombin activity was lower, and PT was slightly more prolonged in the ICU patients (regardless of the normal ranges of both parameters in both groups), whereas D-dimer levels were remarkably higher among the ICU patients.

**Figure 1:**
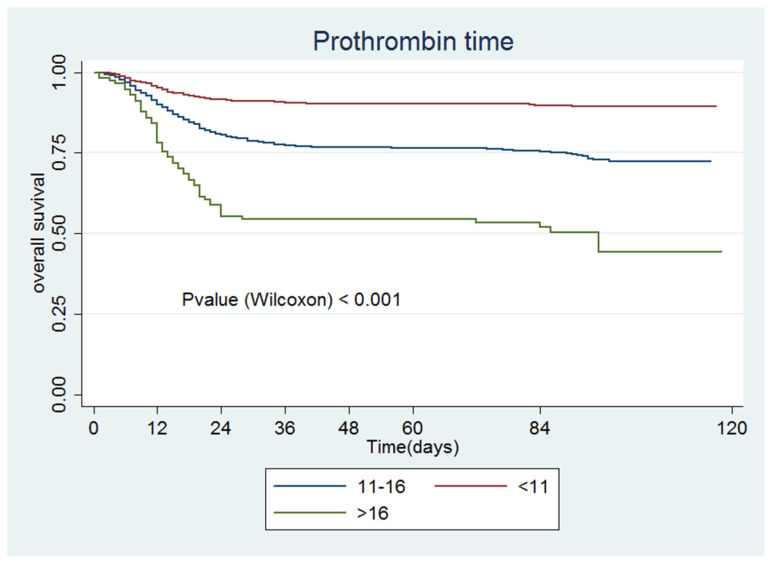
Prothrombin activity.

**Figure 2:**
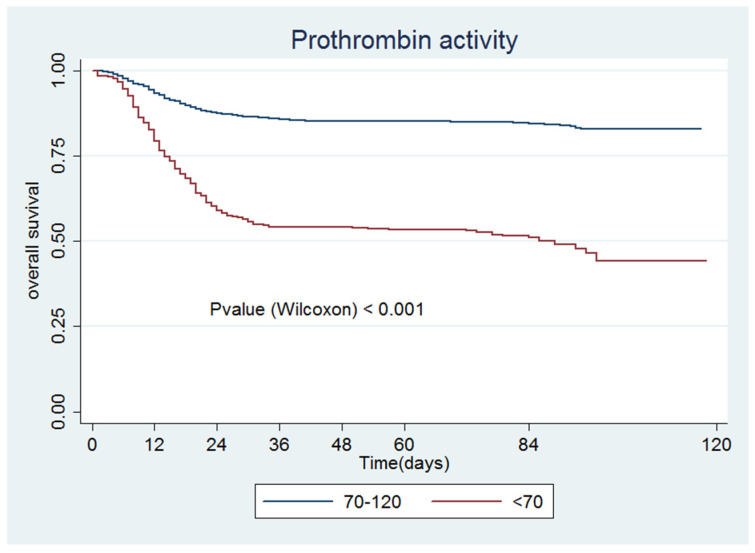
Prothrombin activity.

**Figure 3:**
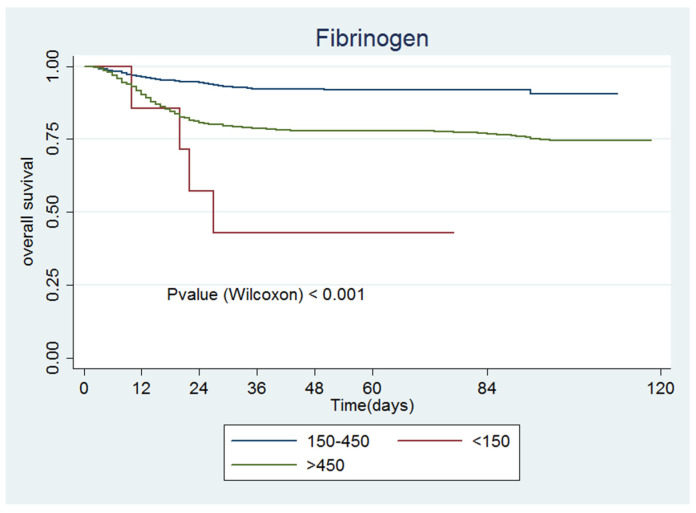
Fibrinogen.

**Figure 4:**
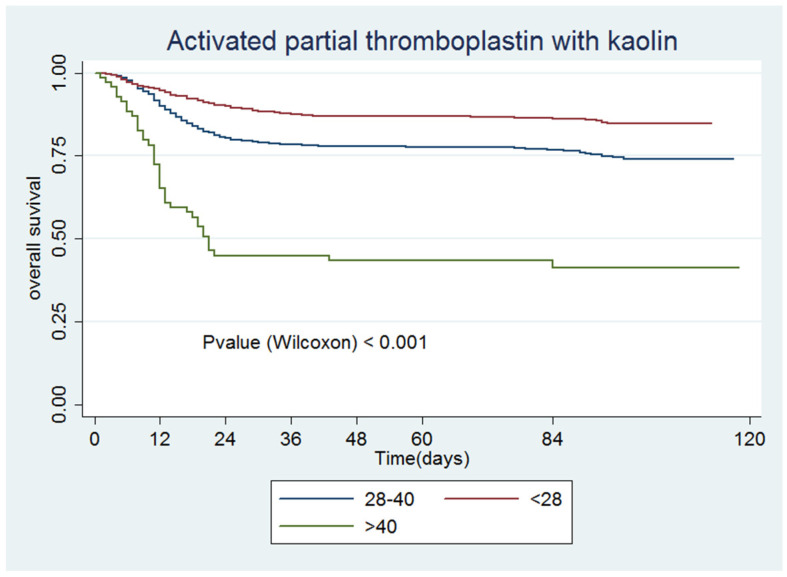
Activated partial thromboplastin time with kaolin.

**Figure 5:**
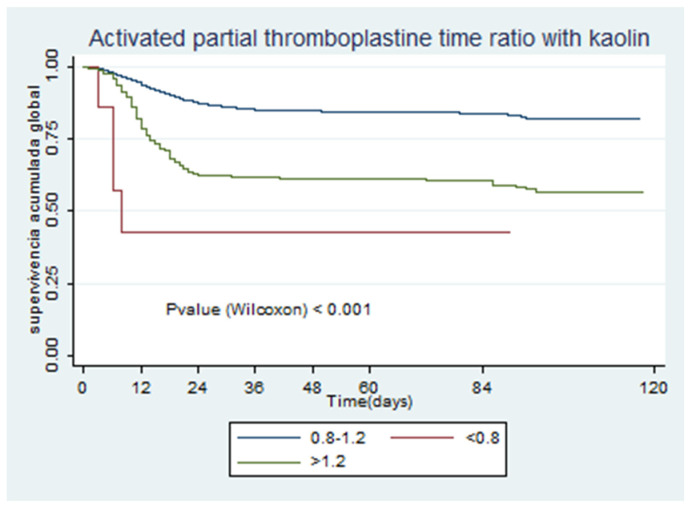
Activated partial thromboplastin time ratio with kaolin.

**Figure 6:**
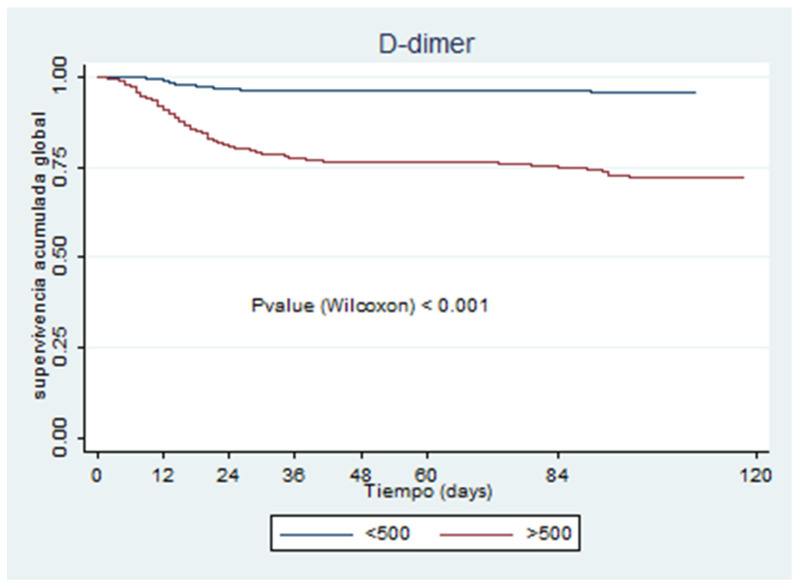
D-dimer.

Figure 1, Figure 2, Figure 3, Figure 4, Figure 5 and Figure 6 show the survival curves according to the cut-off points of the coagulation parameters and for D-dimer, as well as the *p*-values based on the Wilcoxon test. A prolonged prothrombin time (>16 s) was associated with a higher probability of death. Similarly, prolonged aPTT with kaolin (>40 s) was also associated with a higher mortality rate.

Table 4 presents the univariate and multivariate Cox models. All of the coagulation parameters were associated with mortality, given that the values were separated from the normal ranges. Only PT values <11 s and aPTT with kaolin values <28 s were protective against death.

**Figure 7:**
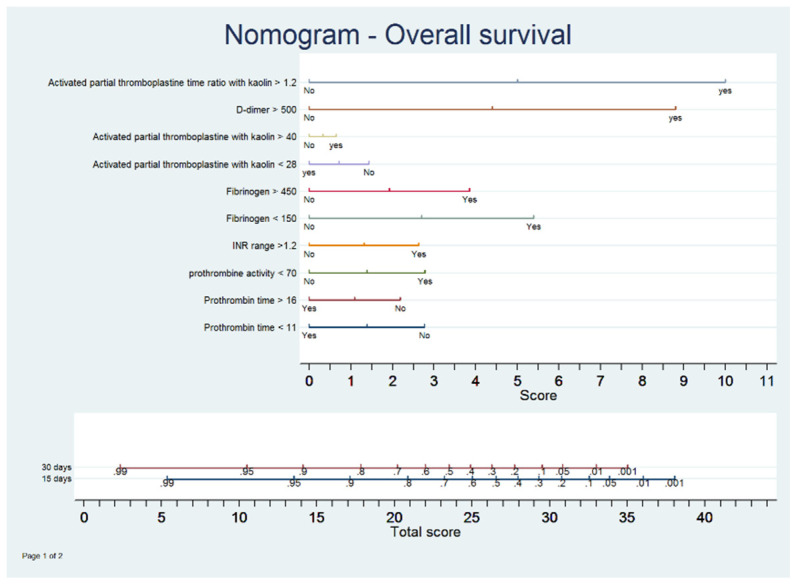
Nomogram.

Figure 7 presents the multivariate model in a nomogram that represents the likelihood of death at 15 and 30 days based on a scale from 0 to 40. Using this scale, we could observe a strong association between each of the coagulation parameters and mortality. The figure shows that an aPTT ratio with kaolin >40 was already associated with a higher score (with practically 10 out of 40 points), followed by D-dimer with 9 out of 40 points. We could also see that for scores >25 points, the 15-day and 30-day survival rates were approximately 60% and 38%, respectively.

## 4. Discussion

The aim of this study was to evaluate the relationship between coagulation abnormalities and prognosis (need for ICU admission, survival, and death) in a cohort of 3581 COVID-19 patients from a tertiary reference hospital in Madrid, Spain. Our study’s strength was its large sample size and a large number of study variables, which provided confidence in the results, consolidating the knowledge on this study’s objective. Although the cumulative differences in the measured parameters were not always striking (some of them moving within the normal range), the figures clearly show that gradual differences in each parameter were associated with mortality (e.g., prothrombin time ratio <70%, D-dimer levels >500 ng/mL).

From the sociodemographic results, we could see that 19.80% of the admissions consisted of healthcare workers. Other studies have reported infection rates among professionals of 3–29%. These findings [17,18] are important considerations due to the current shortage of health professionals for combating the pandemic, which could jeopardize the effectiveness of the health system response and could be exacerbated by the isolation of non-COVID-positive practitioners as preventive measures. [19]. This situation also indirectly leads to exhaustion among the other active workers. A recent study suggested that up to 3% of health workers could be asymptomatically positive, with the consequent risk for other health workers, patients, and the community. For this reason, limiting nosocomial transmission and performing diagnostic tests in the professional field is advisable to better control the disease [20].

The high mortality rate found in our study (approximately 19%) contrasted with the low ICU admission rate (5.08%). During the peak of the pandemic in Spain, a number of patients were declared non-recoverable and, therefore, not eligible for ICU admission, explaining the contrast between the two findings. This situation was not exclusive to Spain; other countries with very high numbers of COVID-19 admissions and with peaks of severe healthcare stress (such as Italy and China) applied similar practices to reduce the stress on the ICU [21].

This study’s main findings were consistent with the poor prognosis associated with abnormal coagulation parameters in COVID-19 patients indicated by other reports. D-dimer levels were more than 3-fold higher in the ICU patients than in those who did not require ICU admission (4190.0 (2347.12–9735.0) vs. 720.0 (410.0–1452.3); *p* < 0.001). Huang et al. [22] reported 5-fold higher D-dimer levels among ICU patients, demonstrating that D-dimer levels were associated with poorer outcomes. In our study, non-survivors showed a more than 3.5-fold increase in D-dimer levels compared with the survivors (2329–1086.12–5670.4) vs. 635.5 (385.5–1194.87); *p* < 0.001). Wang et al. [23] demonstrated that D-dimer levels in non-survivors reached >1000 mg/dL before death. The univariate and multivariate models showed that D-dimer elevation statistically increased the mortality risk (7.03–95% CI 4.93–10.02) and 5.81 (CI 4.05–8.33); *p* < 0.001), which was similar to that reported by Yao et al., who found that D-dimer elevation at admission increased the severity of COVID-19 and was related to a high risk of mortality (OR, 10.17; 95% CI 1.10–94.38; *p* = 0.041) [4]. A recent meta-analysis that included 16 retrospective and 2 prospective studies reported a significant difference in D-dimer levels between survivors and non-survivors, showing an excess risk of up to 4-fold higher in patients with high D-dimer levels, findings that were lower than those of our study. The meta-analysis also concluded that the disease severity was related to medium to high D-dimer levels [24]. In contrast to DIC, which is associated with low platelet counts, elevated D-dimer levels, and low fibrinogen levels, our results supported the assertion that the abnormal coagulation parameters observed in COVID-19 could be different from those in DIC [6]. For instance, our results showed that ICU and non-ICU patients had fibrinogen levels >450 g/dL (681.0 [423.0–882.0] vs. 599.8 (432.5–788.6), *p* = 0.054), as did the survivors and non-survivors (572.75–417.00–758.00) vs. 748.5 (557.0–960.8), *p* < 0.001); however, the fibrinogen levels were higher (with statistical significance) among the non-survivors, thereby indicating that the pathophysiological changes behind these levels are mostly driven by inflammation than by consumption, which is why typical DIC as consumption is not a frequent feature of COVID-19.

Based on the patients’ medical history and disease progression, it is clear that coagulopathies are frequent events in COVID-19 disease. The extremely high D-dimer levels found in this disease and the high fibrinolytic activity could be due to the body’s attempts to eliminate fibrin and necrotic tissue from the lung parenchyma [25,26]. Thromboprophylaxis is, therefore, frequently employed to prevent complications, such as deep vein thrombosis and PE. High D-dimer levels (>1.0 μg/mL) have been associated with deep vein thrombosis in patients not admitted to the ICU, despite the administration of thromboprophylaxis, thereby suggesting the need for prospectively considering aggressive doses of heparin [26]. Considering the results of the univariate model, the mortality risk was higher with levels <150 mg/dL (8.47; 95% CI 3.08–23.26; *p* < 0.001), and the Kaplan–Meier curve (Figure 3) showed that patients with lower fibrinogen levels had a lower survival rate.

Our study has a number of limitations. We excluded a number of patients due to incomplete documentation or a lack of laboratory results. However, our study’s findings were consistent with those of Tang et al. [14], who analyzed 183 patients with novel coronavirus pneumonia and found higher fibrinogen levels among those who did not survive but had sudden low fibrinogen levels shortly before death. A study similar to ours conducted in China with 113 COVID-19 patients obtained similar results, with elevated fibrinogen and D-dimer levels in critically ill patients [27].

## 5. Conclusions

The increase in coagulation parameters could be an efficient measure for predicting the prognosis and improving the clinical management of patients with COVID-19. D-dimer and fibrinogen levels have been clearly shown as predictors of mortality.

## Figures and Tables

**Table 1:** Cohort’s Demographic Characteristics.

Characteristic	
Sex, *n* (%)	
Male	1725 (48.94)
Female	1800 (51.06)
Median age, years (IQR)	62 (47–78)
Healthcare workers, *n* (%)	668 (19.80)
Housing, *n* (%)	
Uncrowded house conditions	3163 (90.60)
Nursing homes	314 (8.99)
Shelter residences	13 (0.37)
Prison	1 (0.03)
Direct/close contact with a confirmed COVID-19 patient, *n* (%)	554 (17.09)
Suspected nosocomial transmission, *n* (%)	1064 (30.56)
Functional dependence, *n* (%)	
Dependence in daily activities	252 (7.44)
Partial dependence in daily activities	190 (5.61)
Independence in daily activities	2943 (86.94)
Severity scales, score (range)	
CURB 65	1 (0–2)
Fine	2 (1–4)
Q–SOFA	0 (0–1)
SOFA	0 (0–1)
PSI	1 (0–4)
Oxygen therapy, *n* (%)	2178 (63.22)
Delivery methods of oxygen therapy, *n* (%)	
Venturi mask	178 (8.18)
Simple face mask	14 (0.64)
Nasal cannula/Nasal prongs	1170 (53.79)
Mask with an oxygen reservoir bag	567 (26.07)
Non-invasive mechanical ventilation	109 (5.01)
Invasive mechanical ventilation	137 (6.30)
Prone position, *n* (%)	188 (6.43)
Positive and expiratory pressure	10.50 (9.50–14.00)
Inspired positive airway pressure	18.00 (14.00–40.00)
Respiratory frequency, bpm	18.00 (18.00–20.00)
ICU admission, *n* (%)	173 (5.08)

Abbreviations: ICU, intensive care unit; IQR, interquartile range; PSI, pneumonia severity index; SOFA, Sepsis-related Organ Failure Assessment; Q–SOFA, quick SOFA.

**Table 2:** Blood Coagulation Parameters between Survivors and Non-survivors.

	Survivors	Non-Survivors	
	*n* = 2731	*n* = 642	*p*
Prothrombin activity, % (IQR)	95.25 (87.00–104.00)	82.5 (67.0–95.0)	<0.001
Fibrinogen, mg/dL (IQR)	572.75 (417.00–758.00)	748.5 (557.0–960.8)	<0.001
INR	1.0 (1.0–1.1)	1.1 (1.0–1.2)	<0.001
Prothrombin time, s (IQR)	11.0 (10.6–11.4)	11.65 (11.03–12.70)	<0.001
D-dimer, ng/mL (IQR)	635.5 (385.5–1194.87)	2329 (1086.12–5670.4)	<0.001
Partial thromboplastin time with kaolin, s (IQR)	27.8 (26.25–29.60)	29.3 (27.0–32.2)	<0.001
Activated partial thromboplastin time ratio with kaolin, s (IQR)	1.04 (0.98–1.11)	1.1 (1.01–1.21)	<0.001

Abbreviations: INR, international normalized ratio; IQR, interquartile range.

**Table 3:** Intensive Care Unit Admissions.

Variable	No (*n* = 3420)	Yes (*n* = 161)	*p*
Prothrombin activity, % (IQR)	93.5 (83.0–103.5)	87.0 (76.0–98.0) %	<0.001
Fibrinogen, mg/dL (IQR)	599.8 (432.5–788.6)	681.0 (423.0–882.0)	0.054
INR, *n* (IQR)	1.0 (1.0–1.1)	1.1 (1.0–1.1)	<0.001
Prothrombin time, s (IQR)	11.1 (10.7–11.6)	11.4 (10.9–12.0)	<0.001
D-dimer, ng/mL (IQR)	720.0 (410.0–1452.3)	4190.0 (2347.12–9735.0)	<0.001
Functional fibrinogen, mg/dL (IQR)	101.0 (74.8–414.0)	78.5 (72.6–88.0)	0.176
Partial thromboplastin time with kaolin, s (IQR)	28.0 (26.4–30.0)	27.95 (26.5–29.8)	0.564
Activated partial thromboplastin time ratio with kaolin, mg/dL (IQR)	1.05 (0.99–1.12)	1.05 (0.99–1.12)	0.592

Abbreviations: INR, international normalized ratio; IQR, interquartile range.

**Table 4:** Univariate and Multivariate Cox Models.

	Univariate Model			Multivariate Model		
	HR	95% CI	*p*	HR	95% CI	*p*
Prothrombin activity						
<70	3.91	3.27–4.68	<0.001	1.74	1.21–2.51	0.003
70–120	Ref			Ref		
Fibrinogen						
<150	8.47	3.08–23.26	<0.001	2.93	1.02–8.39	0.044
150–450	Ref			Ref		
>450	3.03	2.34–3.92	<0.001	2.16	1.58–2.95	<0.001
INR						
0.8–1.2	Ref			Ref		
>1.2	4.17	3.43–5.07	<0.001	1.69	1.11–2.57	0.014
Prothrombin time, s						
<11	0.37	0.30–0.45	<0.001	0.57	0.45–0.73	<0.001
11–16	Ref			Ref		
>16	2.31	1.74–3.05	<0.001	0.64	0.38–1.07	<0.001
D-dimer						
≤500	Ref			Ref		
>500	7.03	4.93–10.02	<0.001	5.81	4.05–8.33	<0.001
Activated partial thromboplastin time with kaolin, s						
<28	0.56	0.47–0.66	<0.001	0.75	0.60–0.93	0.010
28–40	Ref			Ref		
>40	3.32	2.39–4.61	<0.001	1.13	0.64–1.99	0.652
Activated partial thromboplastin time ratio with kaolin						
<0.8	6.54	2.44–17.51	<0.001	7.37	1.01–53.50	0.048
0.8–1.2	Ref	Ref				
>1.2	2.91	2.41–3.51	<0.001	1.65	1.25–2.18	<0.001

Abbreviations: CI, confidence interval; HR, hazard ratio; INR, international normalized ratio.
